# GNG5 is an unfavourable independent prognostic indicator of gliomas

**DOI:** 10.1111/jcmm.15923

**Published:** 2020-09-30

**Authors:** Biao Yang, Zhen‐Yuan Han, Wen‐Juan Wang, Yan‐Bin Ma, Sheng‐Hua Chu

**Affiliations:** ^1^ Department of Neurosurgery Shanghai Ninth People's Hospital Affiliated to Shanghai Jiao Tong University School of Medicine Shanghai China; ^2^ Department of Neurosurgery Huashan Hospital of Fudan University Shanghai China; ^3^ Department of Oral Pathology Shanghai Ninth People’s Hospital Affiliated to Shanghai Jiao Tong University School of Medicine Shanghai China; ^4^ Department of Orthopaedic Surgery Shanghai Key Laboratory of Orthopaedic Implants Shanghai Ninth People’s Hospital Shanghai Jiao Tong University School of Medicine Shanghai China

**Keywords:** glioma, GNG5, prognosis, progression, subtype

## Abstract

Gliomas are the most common primary brain tumours, and glioblastomas (GBMs) are subgrouped into four distinct molecular subtypes. This study aimed to identify the potential gene related to glioma progression. Weighted gene co‐expression network analysis (WGCNA) was used to explore the related gene. Correlation, ROC, survival and Cox regression analyses were performed. Blue module was strongly associated with WHO grade (*r* = .65, *P* = 1e‐19). GNG5 in gliomas was overexpressed compared with normal samples and associated with clinicopathologic characteristics. GNG5 was frequent in Mesenchymal subtype and lowly expressed in Proneural subtype of GBMs. Survival and Cox regression analyses showed that glioma patients with GNG5 overexpression had shorter survival time, and GNG5 was an independent prognostic indicator of overall survival. Overall, GNG5 expression is closely associated with clinicopathologic characteristics and is an independent prognostic indicator for glioma patients, as well as a promising subtype‐associated biomarker in molecular classification of gliomas.

## 1 INTRODUCTION

Gliomas in adult are the most common brain tumours with poor patient survival rates and limited life expectancy, which account for more than 70% of malignant brain tumours.^1^ Glioblastomas (GBMs) are the most malignant tumours in gliomas and the median survival is <2 years with the current standard therapy of maximal surgery resection, followed by combined chemoradiotherapy.^2^ On the basis of 2007 WHO classification of CNS tumours, the updated 2016 WHO classification added molecular characteristics to diagnosis criteria, including 1p/19q codeletion and isocitrate dehydrogenase (IDH) mutation.^3^ According to their molecular signatures, the TCGA classified GBMs into four subtypes: Classical, Mesenchymal (Mes), Neural and Proneural (PN).^4^ The classification improves the treatment and prognosis of gliomas. However, more genes related to diagnosis and prognosis has not yet been fully discovered.

Reportedly, G protein subunit gamma 5 (GNG5) gene encoding the G‐γ5 subtype was highly expressed in neural progenitor cells in both adult brain and embryonic, and its presence in focal adhesions was important to regulate cellular adhesion, proliferation and migration.^5^ The latest literature showed that GNG5 can regulate chondrocyte apoptosis and cartilage degradation.^6^ However, the potential value of GNG5 in gliomas is not entirely clear.

In this study, the integrated bioinformatics analyses including weighted gene co‐expression network analysis (WGCNA) and functional enrichment analysis were performed to extract the potential gene. Additionally, correlation, survival and Cox regression analyses were conducted to explore its clinical role in gliomas.

## 2 MATERIALS AND METHODS

### 2.1 Downloading and pre‐processing of data, and usage of databases

The glioma datasets (including GSE4271, GSE4290 and GSE68848) were obtained from Gene Expression Omnibus (GEO) database. In addition, low‐grade glioma (LGG) and GBM were downloaded from The Cancer Genome Atlas (TCGA). Moreover, three mRNA including mRNA‐array_301, mRNAseq_325 and mRNAseq_693 were obtained from Chinese Glioma Genome Atlas (CGGA). Additionally, comparison statistical analysis with the criteria of *P* < .05 and fold change (FC) >1.5 was performed using Oncomine.^7^ GEPIA2 was used for correlation and survival analyses.^8^

### 2.2 Identification of differentially expressed genes (DEGs)

Expression profile of GSE4290 with 157 glioma and 23 normal brain samples was pre‐processed and subsequently performed to identify DEGs with the cut‐off criteria of adjusted *P* < .01 and |log2FC| > 1.

### 2.3 Construction of WGCNA

Based on DEGs from GSE4290, weighted gene co‐expression network analysis was performed using WGCNA package in R 3.5.0.^9^ An appropriate soft‐thresholding value was analysed to meet the scale‐free topology. The relationships between co‐expression modules and clinical information were calculated. Top 20 mRNAs with highest intra‐modular connectivity in the interested module were selected as hub genes.

### 2.4 Functional annotation for the blue module

All mRNAs in the blue module were uploaded to DAVID v6.8 for functional analyses, including Gene Ontology (GO) and Kyoto Encyclopedia of Genes and Genomes (KEGG) pathway analyses.^10^, ^11^ The terms with *P* < .05 were considered to be significant.

### 2.5 Statistical analyses

Statistical analyses were performed by using SPSS 21.0, GraphPad Prism 7.0 and R 3.5.0. ROC curves, univariate and multivariate Cox regression analyses and survival analyses were analysed by R 3.5.0. The difference with *P* < .05 was considered to be statistically significant.

## 3 RESULTS

### 3.1 Identification of DEGs

**Figure 1:**
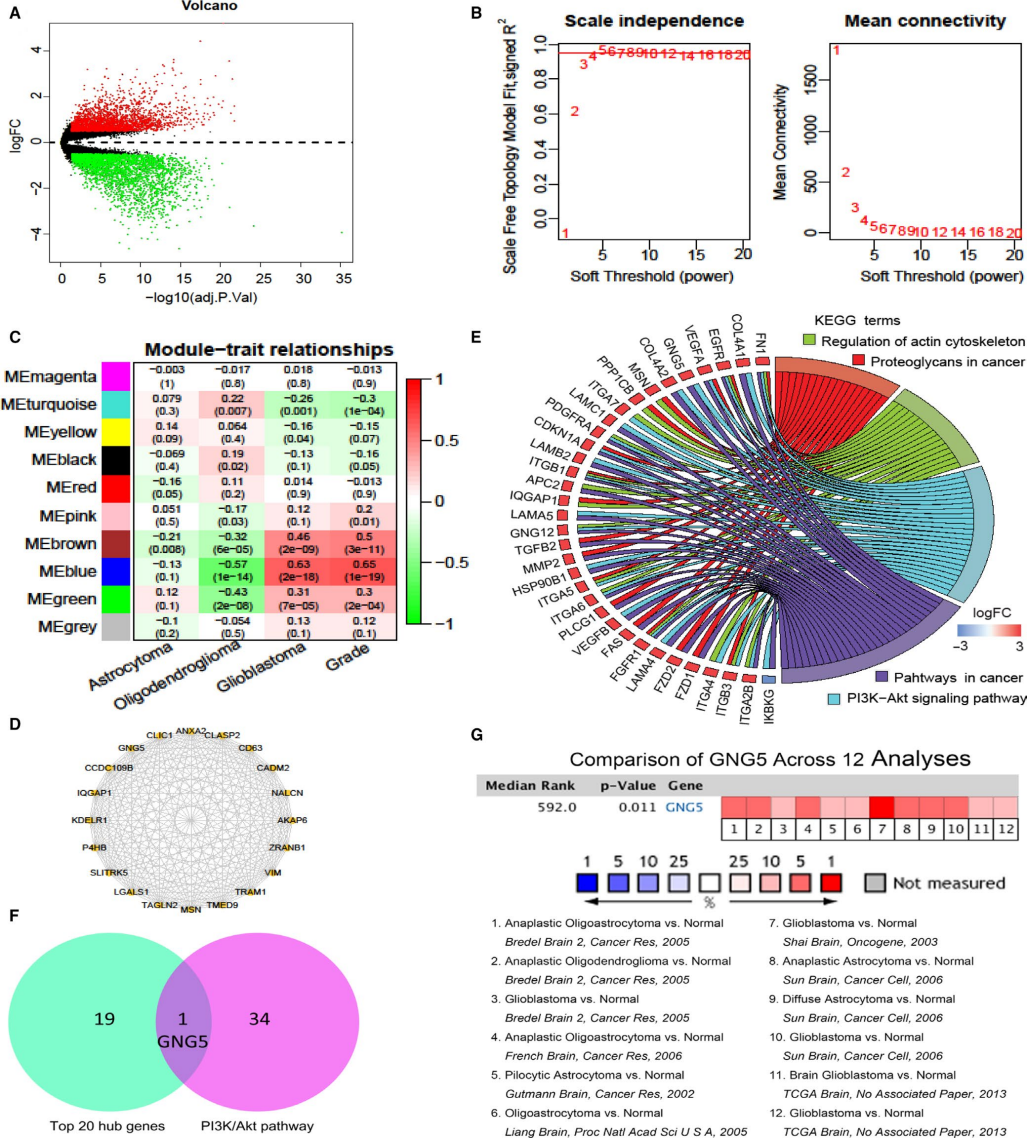
Identification of GNG5 by using weighted gene co‐expression network analysis. A, Volcano plot of DEGs between glioma and normal samples among GSE4290 with the criteria of *P* < .05 and |log2FC| > 1. B, Analyses of network topology for various soft‐thresholding powers. C, Module‐trait relationships. D, Top 20 hub genes in blue module. E, The KEGG functional enrichment analyses of all genes in blue module. F, Venn diagram of the top 20 hub genes and the genes of the PI3K/Akt signalling pathway. GNG5 was the only common gene between the two cohorts. G, The comparison analyses across 12 datasets from the Oncomine database showed that GNG5 in gliomas was significantly up‐regulated compared with normal samples with the cut‐off threshold of *P* < .05 and fold change > 1.5. DEGs, differentially expressed genes; FC, fold change; KEGG, Kyoto Encyclopedia of genes and genomes

The dataset GSE4290 with glioma samples carrying genetic expression and WHO grade were obtained. A total of 8288 DEGs, including 2711 up‐regulated and 3386 down‐regulated DEGs, were identified between 157 glioma and 23 normal brain samples with the criteria of *P* < .05 and |log2FC| > 1 (Figure 1A).

### 3.2 Construction of WGCNA, identification of blue module and selection of hub genes

The obtained genetic profile of 8288 DEGs among GSE4290 was performed for WGCNA. No sample outlier was extracted out after quantity assessment, and the scale‐free topology was achieved when the soft‐thresholding power was 5 (Figure 1B). In total, 9 co‐expression modules were identified and other genes were added into the grey module (Figure 1C). The trait heat map was shown in Figure 1C and the blue module which had the strongest correlation with WHO grade (*r* = .65, *P* = 1e‐19) was selected as the interested module. Furthermore, based on the intra‐modular connectivity of all genes in the blue module, top 20 genes were identified as the hub genes for the next analyses (Figure 1D).

### 3.3 Functional enrichment analyses of the blue module

After functional annotations for 1245 genes in the blue module, GO results showed that blue module was associated with phospholipase inhibitor activity, focal adhesion and so on, which indicated the biological significance and phosphorylation role of tumour development (Table S1). In addition, KEGG results showed that genes of the co‐expression module were correlated with proteoglycans in cancer, regulation of actin cytoskeleton, PI3K/Akt signalling pathway and pathway in cancer (Figure 1E).

### 3.4 Identification of the potential signature gene GNG5

PI3K/Akt signalling pathway, which contained 35 genes in this study, played critical roles in the pathogenesis of tumours, especially glioma. Furthermore, a protein‐coding gene, GNG5, was identified by merging of these 35 genes from PI3K/Akt signalling pathway and top 20 hub genes (Figure 1F). Therefore, GNG5 was selected a potential signature gene in glioma diagnosis and prognosis for the next analyses.

### 3.5 Overexpressed GNG5 was associated with clinicopathological characteristics and molecular classification in gliomas

To better understand the GNG5 expression patterns in brain and CNS tumours, the Oncomine database was applied to present the expression features of GNG5 which indicated that the GNG5 was significantly up‐regulated in brain and CNS tumours compared to the normal tissues (Figure 1G, *P* < .05 and FC > 1.5).

**Figure 2:**
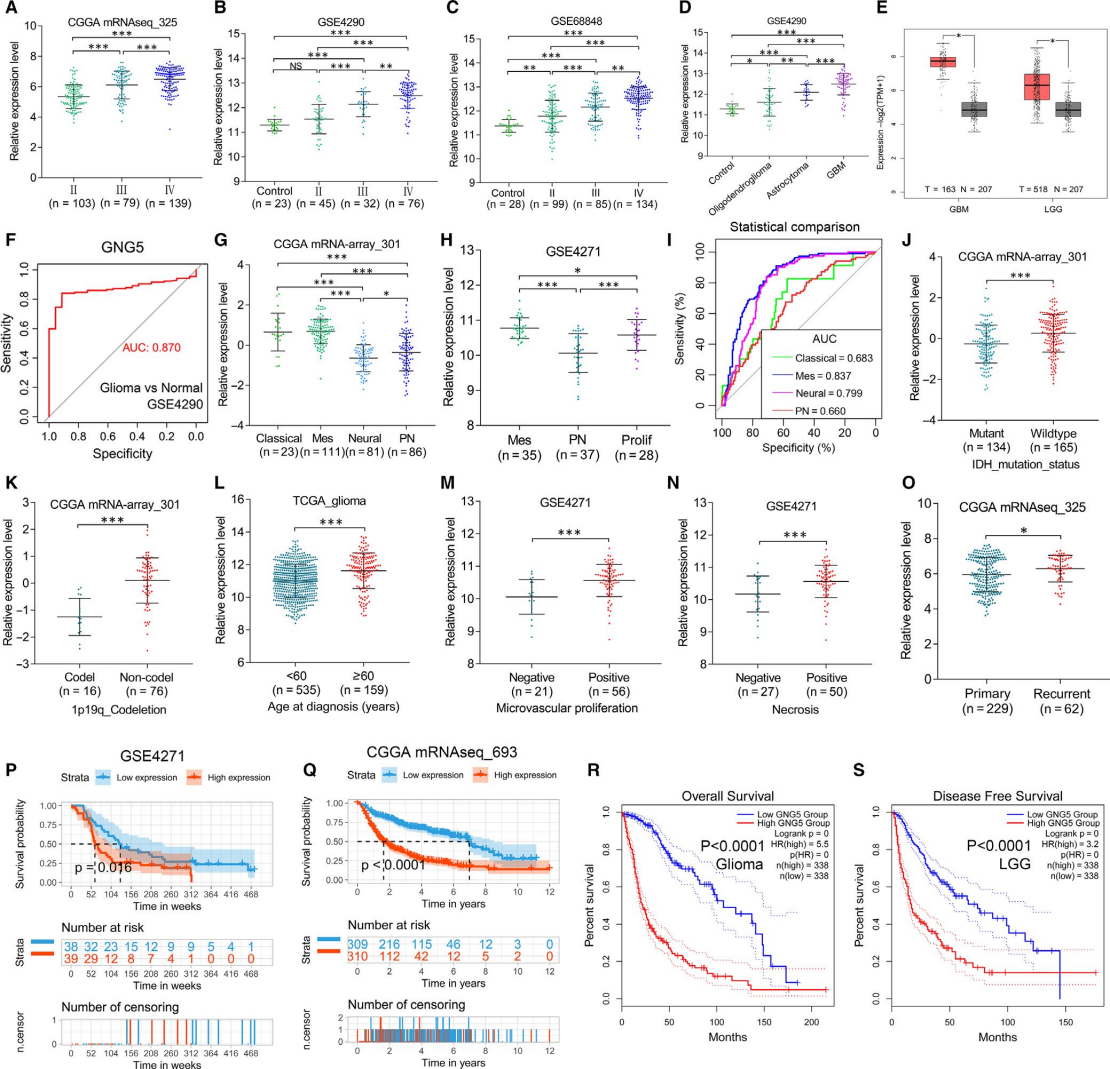
GNG5 expression contributes to malignant procession and has clinical prognostic impact in gliomas A‐C, GNG5 expression in gliomas was up‐regulated with advanced WHO grades in CGGA mRNAseq_325, GSE4290 and GSE68848, respectively. D, The expression of GNG5 was analysed in GSE4290. E, GNG5 in GBM and LGG samples was overexpressed compared with the normal brain samples using online GEPIA2. F, ROC analysis showed that GNG5 was an effective potential signature gene of diagnosis prediction of the glioma histology in GSE4290. G and H, The expression of GNG5 in different subtypes of gliomas in the two datasets included CGGA mRNA‐array_301 and GSE4271. I, ROC analysis in CGGA mRNA‐array_301 showed that the role of GNG5 in molecular classification in gliomas. J and K, The relationships between GNG5 expression and molecular characteristics (IDH1 mutation and 1p/19q codeletion status) were analysed in the dataset CGGA mRNA‐array_301. L‐N, The relationships between GNG5 expression and clinical features (including age, microvascular proliferation and necrosis) were analysed. O, Recurrent glioma samples have higher expression levels of GNG5 than primary samples in CGGA mRNA‐array_325. P and Q, The Kaplan‐Meier plots of GNG5 were in GSE4271 and CGGA mRNAseq_693, respectively. The horizontal axis indicates time in years, and the vertical axis represents the probability of surviving. R and S, The glioma patients with high GNG5 expression have shorter overall survival and disease‐free survival compared with the low GNG5 group using the tool GEPIA2 (*P* < .05). Red lines indicate the group with the high GNG5 expression and blue lines represent the group with the low GNG5 expression

Next, similar to the result of the positive correlation between WHO grade and blue module in WGCNA, GNG5 expression was positively associated with WHO grade of gliomas in the datasets, including CGGA mRNAseq_325, GSE4290 and GSE68848, respectively (Figure 2A‐C). Subsequently, GNG5 expression was up‐regulated in histology order of normal brain, oligodendroglioma, astrocytoma and GBM (Figure 2D). GNG5 was also up‐regulated in GBM and LGG samples compared with normal samples (Figure 2E). Furthermore, ROC analysis showed that GNG5 could be an effective potential signature gene of diagnosis prediction between glioma and normal samples in GSE4290 (AUC = 0.870; Figure 2F).

More importantly, GNG5 had significantly different expression in molecular subtypes in different datasets, including CGGA mRNA‐array_301 and GSE4271, respectively (Figure 2G,H). And ROC analysis showed that GNG5 was a good signature gene to identify different molecular classification (Figure 2I). In addition, GNG5 expression was strongly related to important molecular characteristics of gliomas, such as IDH mutation and 1p/19q codeletion status in CGGA mRNA‐array_301 (Figure 2J,K).

Moreover, it was observed that the overexpression of GNG5 had a positive correlation with the factors including older age (≥60 years), microvascular proliferation, necrosis and recurrence (Figure 2L‐O). Collectively, these findings indicate that overexpressed GNG5 is associated clinicopathological characteristics of glioma patients.

### 3.6 GNG5 could function as an independent and inferior factor on glioma prognosis

According to the median of GNG5 expression, glioma samples were divided into two groups: low GNG5 expression group and high GNG5 expression group. If the total sample number is odd, the high expression group has one more sample than the low expression group. The survival result in explicitly illustrated that 39 gliomas with GNG5 overexpression have shorter overall survival compared with the 38 samples with GNG5 low expression (*P* = .016; Figure 2P). Also, similar results were obtained from another dataset CGGA mRNAseq_693 (*P* < .0001; Figure 2Q). Glioma patients with high GNG5 expression had shorter overall survival (OS; *P* < .0001; Figure 2R) and disease‐free survival (DFS; *P* < .0001; Figure 2S) compared with the low GNG5 group using the GEPIA2. Cox regression analyses were conducted in TCGA_glioma. As shown in Table S2, multivariate Cox regression analyses in the two datasets TCGA_glioma and TCGA_LGG similarly revealed that GNG5 expression as well as age and WHO grade was an independent prognostic factor of OS. Taken together, these results reveal that GNG5 expression is strongly associated with a poor prognosis and serves an independent prognostic factor of OS for gliomas.

## 4 DISCUSSION

Gliomas are the most common primary tumours of brain and spinal cord, and patients still have low patient survival rates and limited life expectancy.^1^ In this study, a subtype‐associated independent prognosis factor, GNG5, was identified in gliomas by using an integrated bioinformatics method and served as an important role in glioma pathogenesis.

Moreover, GNG5 expression was found to be associated with WHO grade, age, histology, recurrence, necrosis and microvascular proliferation. And these factors are always associated with prognosis and survival time of gliomas, which were also verified by Cox regression analyses in this study. Subsequently, survival analyses showed that gliomas with GNG5 overexpression had shorter OS and DFS time compared with low GNG5 gliomas. More importantly, multivariate Cox regression analyses illustrated that GNG5 could function as an independent prognostic factor of OS. Taken together, GNG5 is associated with clinical features and acts an independent prognostic indicator of OS in gliomas, suggesting that GNG5 might play a critical role in tumorigenesis and be a potential gene of diagnosis, treatment and prognosis for glioma patients.

In 2016 molecular characteristics were added into the WHO glioma classification, and some molecular subtypes were identified.^4^, ^12^ Mes subtype patients were positively associated with the proportion of necrosis in GBMs and PN subtype patients have a better prognosis compared with other subtypes.^4^ GNG5 was found to trend to be overexpressed in Mes subtype and downexpressed in PN subtype compared with other two subtypes. Patients with GBMs or anaplastic astrocytomas carrying an IDH mutation had a longer median survival time than the same patients with IDH wildtype.^13^ Likewise, our study showed that GNG5 expression was down‐regulated in gliomas with IDH mutation and glioma patients with low GNG5 expression had a longer OS time compared with the high GNG5 expression group. Similarly, glioma patients with 1p/19q codeletion carried an IDH mutation,^14^ and in our study that glioma patients carrying 1p/19q codeletion had a lower expression level of GNG5. Thus, GNG5 might be a potential gene of molecular classification in gliomas.

GNG5 was found to be involved in pathogenesis of diseases in mice,^5^ but its potential value in diseases are yet to be discovered. In this study, KEGG analyses results of the blue module showed that GNG5 was involved in PI3K/Akt signalling pathway and pathways in cancer. These suggested that GNG5 might regulate glioma growth by activating PI3K/Akt signalling pathway. PI3K activated its downstream molecule Akt, which lead to the phosphorylation and ultimately promotes tumour growth. This pathway was reported to serve as a critical role in many biological processes of tumours such as proliferation, migration and survival.^15^

In conclusion, this study demonstrated that GNG5 was overexpressed in gliomas and significantly associated with clinicopathologic characteristics and molecular subtypes, suggesting that it was a subtype‐associated gene in the molecular classification of gliomas. GNG5 was also found to serve as an independent prognostic indicator for glioma patients.

## CONFLICTS OF INTEREST

The authors declare that they have no conflicts of interests.

## AUTHOR CONTRIBUTIONS

**Biao Yang:** Methodology (equal); Visualization (equal); Writing‐original draft (equal). **Zhen‐Yuan Han:** Formal analysis (equal); Methodology (equal); Writing‐original draft (equal). **Wen‐Juan Wang:** Formal analysis (equal); Writing‐original draft (equal); Writing‐review & editing (equal). **Yan‐Bin Ma:** Software (equal); Visualization (equal). **Sheng‐Hua Chu:** Conceptualization (equal); Funding acquisition (equal).

## Supporting information

Table S1

Table S2

## Data Availability

The data that support the findings of this study were derived from the following resources available in the public domain: Gene Expression Omnibus, The Cancer Genome Atlas, and the Chinese Glioma Genome Altas.
