# Supplementary material for: GNG5 is an unfavourable independent prognostic indicator of gliomas
Source: J Cell Mol Med. 2020 Sep 30;24(21):12873–8. doi: 10.1111/jcmm.15923 (PMC7686969; doi:10.1111/jcmm.15923)
Supplement: Supplementary file 1 — Table S1 [file JCMM-24-12873-s001.docx]

Supplementary Table S1 Top 5 significant GO enrichment annotations of all genes in blue module.

| Category | Term | Count | PValue | FDR |
| --- | --- | --- | --- | --- |
| GOTERM_BP_DIRECT | BP:antigen processing and presentation of peptide antigen via MHC class I | 13 | 7.62E-08 | 1.40E-04 |
| GOTERM_BP_DIRECT | BP:angiogenesis | 36 | 2.29E-07 | 4.21E-04 |
| GOTERM_BP_DIRECT | BP:ER to Golgi vesicle-mediated transport | 28 | 1.30E-06 | 0.002380747 |
| GOTERM_BP_DIRECT | BP:interferon-gamma-mediated signaling pathway | 17 | 4.28E-06 | 0.007857451 |
| GOTERM_BP_DIRECT | BP:platelet degranulation | 19 | 4.57E-05 | 0.083939274 |
| GOTERM_CC_DIRECT | CC:focal adhesion | 68 | 6.65E-15 | 9.93E-12 |
| GOTERM_CC_DIRECT | CC:endoplasmic reticulum membrane | 102 | 8.17E-11 | 1.22E-07 |
| GOTERM_CC_DIRECT | CC:extracellular exosome | 242 | 2.81E-09 | 4.19E-06 |
| GOTERM_CC_DIRECT | CC:membrane | 192 | 8.25E-08 | 1.23E-04 |
| GOTERM_CC_DIRECT | CC:extracellular matrix | 43 | 2.16E-07 | 3.22E-04 |
| GOTERM_MF_DIRECT | MF:protein binding | 659 | 1.54E-16 | 1.78E-13 |
| GOTERM_MF_DIRECT | MF:hydrolase activity, hydrolyzing O-glycosyl compounds | 10 | 4.70E-05 | 0.075432139 |
| GOTERM_MF_DIRECT | MF:protein homodimerization activity | 71 | 8.21E-05 | 0.131646023 |
| GOTERM_MF_DIRECT | MF:phospholipase inhibitor activity | 6 | 2.72E-04 | 0.435953077 |
| GOTERM_MF_DIRECT | MF:aminopeptidase activity | 8 | 8.84E-04 | 1.407907859 |

GO, Gene Ontology; BP, biological process; MF, molecular function; CC, cell component.
