# Supplementary material for: GNG5 is an unfavourable independent prognostic indicator of gliomas
Source: J Cell Mol Med. 2020 Sep 30;24(21):12873–8. doi: 10.1111/jcmm.15923 (PMC7686969; doi:10.1111/jcmm.15923)
Supplement: Supplementary file 2 — Table S2 [file JCMM-24-12873-s002.docx]

Supplementary Table S2. Cox regression analysis of GNG5 expression as a survival predictor of gliomas in TCGA_glioma.

| Parameter | Univariate analysis | | |  | Multivariate analysis | | |
| --- | --- | --- | --- | --- | --- | --- | --- |
|  | P | HR | 95%CI |  | P | HR | 95%CI |
| Age *(≥60 vs. <60 years)* | **P<0.001** | 1.06 | 1.05-1.07 |  | **P<0.001** | 1.05 | 1.03-1.07 |
| Gender *(Male vs. Female)* | **0.047** | 1.28 | 1.00-1.63 |  | 0.583 | 1.13 | 0.72-1.78 |
| Race *(Asian vs. Black or african American vs. White)* | 0.750 | 0.94 | 0.64-1.37 |  | NA | NA | NA |
| WHO grade *(II vs. III vs. IV)* | **P<0.001** | 4.50 | 3.69-5.49 |  | **P<0.001** | 2.70 | 1.50-4.84 |
| Histology  *(Astrocytoma, anaplastic vs. Astrocytoma, NOS vs. GBM vs. Mixed glioma vs. Oligodendroglioma, anaplastic vs. Oligodendroglioma, NOS)* | **P<0.001** | 0.82 | 0.77-0.88 |  | 0.387 | 0.94 | 0.81-1.09 |
| TCGA_subtypes *(Classical vs. Mesenchymal vs. Neural vs. Proneural)* | 0.688 | 0.97 | 0.83-1.13 |  | NA | NA | NA |
| KPS *(≥80 vs. <80 scores)* | **0.044** | 0.57 | 0.33-0.99 |  | 0.233 | 0.69 | 0.37-1.27 |
| Treatment_or_therapy *(Positive vs. Negative)* | **P<0.001** | 1.99 | 1.45-2.73 |  | 0.313 | 0.72 | 0.38-1.37 |
| GNG5 expression *(High vs. low)* | **P<0.001** | 2.43 | 2.13-2.77 |  | **P<0.001** | 1.91 | 1.40-2.59 |

Note: TCGA, The Cancer Genome Atlas; WHO, World Health Organization; GBM, glioblastoma multiforme; KPS, Karnofsky performance score; NA, not analyze.
